# Relevance Function of Linc-ROR in the Pathogenesis of Cancer

**DOI:** 10.3389/fcell.2020.00696

**Published:** 2020-08-11

**Authors:** Wenjian Chen, Junfa Yang, Hui Fang, Lei Li, Jun Sun

**Affiliations:** ^1^Anhui Provincial Children’s Hospital, Affiliated to Anhui Medical University, Hefei, China; ^2^Key Laboratory of Anti-inflammatory and Immune Medicine, Ministry of Education, Institute of Clinical Pharmacology, Anhui Medical University, Hefei, China; ^3^School of Pharmacy, Anhui Medical University, Hefei, China; ^4^Department of Pharmacology, The Affiliated Hospital of Hangzhou Normal University, Hangzhou, China; ^5^The Affiliated Hospital of Anhui Medical University, Hefei, China

**Keywords:** lncRNAs, ncRNAs, linc-ROR, cancers, biomarker

## Abstract

Long non-coding RNAs (lncRNAs) are the key components of non-coding RNAs (ncRNAs) with a length of 200 nucleotides. They are transcribed from the so-called “dark matter” of the genome. Increasing evidence have shown that lncRNAs play an important role in the pathophysiology of human diseases, particularly in the development and progression of tumors. Linc-ROR, as a new intergenic non-protein coding RNA, has been considered to be a pivotal regulatory factor that affects the occurrence and development of human tumors, including breast cancer (BC), colorectal cancer (CRC), pancreatic cancer (PC), hepatocellular carcinoma (HCC), and so on. Dysregulation of Linc-ROR has been closely related to advanced clinicopathological factors predicting a poor prognosis. Because linc-ROR can regulate cell proliferation, apoptosis, migration, and invasion, it can thus be used as a potential biomarker for patients with tumors and has potential clinical significance as a therapeutic target. This article reviewed the role of linc-ROR in the development of tumors, its related molecular mechanisms, and clinical values.

## Introduction

Cancer is a serious disease that affects human health, being one of the main causes of death all over the worldwide. According to research in 2018, 59% of new tumor cases and 70% of the cancer-associated deaths occurred in low-income and developing countries (Kumar and Sharawat, 2018; Noorolyai et al., 2019). Owing to a shortage in effective screening methods and lack of identification of early symptoms, most patients were already in advanced stages when they were diagnosed with cancer (Bray et al., 2018; Koo et al., 2018). Additionally, some clinical studies have shown that polarity and adhesion of cancer cells was decreased, leading to heir increased mobility and invasion, which is a key step in the development of cancer (Yan et al., 2018). Therefore, studies have shown that the high mobility of cancer cells is the main factor leading to high mortality rates in patients with cancer. Currently, there are many ways employed in the treatment of cancer, including surgery, radiotherapy, chemotherapy, biotherapy and targeted therapy (Nie et al., 2016). However, in the past 5 years, the survival rate of patients with cancers remains dismal (Nakashima, 2018). Therefore, in the process of developing human antitumor strategies, it is particularly important to find new early biomarkers and thus identify potential regulatory mechanisms to improve the survival rate of patients with cancers.

**FIGURE 1:**
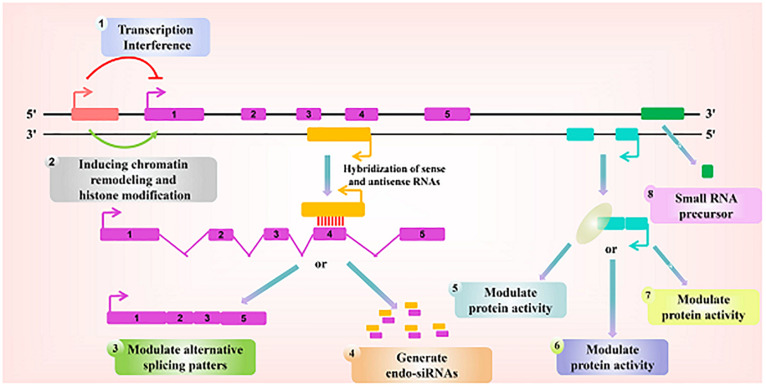
Paradigms for the function of long ncRNAs. Recent studies have identified a variety of regulatory paradigms for the mechanism by which long ncRNAs function, many of which are highlighted here. Transcription from an upstream non-coding promoter (pink) can negatively **(1)** or positively **(2)** affect the expression of the downstream gene (purple) by inhibiting the recruitment of RNA polymerase II or inducing chromatin remodeling, respectively. **(3)** An antisense transcript (orange) is able to hybridize to the overlapping sense transcript (purple) and block the recognition of the splice sites by the spliceosome, thus resulting in an alternatively spliced transcript. **(4)** Alternatively, hybridization of the sense and antisense transcripts can allow Dicer to generate endogenous siRNAs. By binding to specific protein partners, a non-coding transcript (blue) can modulate the activity of the protein **(5)**, serve as a structural component that allows the formation of a larger RNA-protein complex **(6)**, or alter where the protein localizes in the cell **(7)**. **(8)** Long ncRNAs (green) can be processed to yield small RNAs, such as miRNAs, piRNAs, and other less well-characterized classes of small transcripts.

Over the past 2 decades, ncRNAs constitute more than 90% of the RNAs made from the human genome, but most of the > 50,000 known non-coding RNAs (ncRNAs) have been discovered and remain largely unstudied (Bhan et al., 2017; Slack and Chinnaiyan, 2019). Transfer RNA (tRNA) (89%) and ribosomal RNA (rRNA) (8.9%) constitute the majority of ncRNAs, followed in abundance by messenger RNAs (mRNAs) (0.9%). Thus, the remaining ncRNAs, including circular RNA (circRNA), small nuclear RNA (snRNA), small nucleolar RNA (snoRNA), microRNA (miRNA), and long non-coding RNA (lncRNA) together account for ∼1% of total ncRNA. Despite their low abundance, these ncRNAs have been reported to play critical roles in transcription, post-transcriptional processing, and translation such as epigenetics, post-transcriptional regulation, chromatin modification, and regulation of the cell cycle (Huarte, 2015; Kondo et al., 2017; Peng et al., 2017b). Additionally, because ncRNAs can be packaged into extracellular vesicles (EV), including exosomes (Meldolesi, 2018), they have been shown to provide a mechanism for intercellular communication through the transfer of miRNA and lncRNA to recipient cells both locally and systemically (Sun et al., 2018). It is important to note that the expression of ncRNAs, their post-transcriptional modification (particularly lncRNAs), and their subcellular distribution have been shown to be important to when assigning their potential function (Palazzo and Lee, 2015). Recently, next-generation sequencing and bioinformatics technology have revealed that circRNAs play crucial role in diagnosis and prognosis of various diseases (Pamudurti et al., 2017). Briefly, circRNAs are single-stranded transcripts generated by back-splicing (Jeck and Sharpless, 2014), with covalently linked head-to-tail closed loop structures with neither 5′–3′ polarity nor a polyadenylated tail (Memczak et al., 2013), that range in length from a few hundred to thousands of nucleotides, and are widely expressed in mammals, thereby showing higher stability compared to that in linear RNAs (Chen J. et al., 2017), and exhibiting a cell-type- or developmental-stage-specific expression pattern (Barrett and Salzman, 2016; Wang J. J. et al., 2019). Many functions of circRNAs have also been identified, including their role as miRNA sponges, binding to RNA-binding proteins and protein decoys, and functioning as regulators of transcription (Hansen et al., 2013; Du et al., 2016; Yang Y. et al., 2018). Interestingly, many circRNAs have been shown to be dysregulated in pathophysiological processes, and circRNAs are known to regulate the expression of gene by acting as miRNA sponges, in a mechanism that is termed as competitive endogenous RNA (ceRNA) mechanism (Zheng et al., 2016; Wang J. J. et al., 2019). For example, circMTO1 have been demonstrated to harbor conventional miRNA binding sites and has been identified as an inhibitor of miRNA-9 in hepatocellular carcinoma (HCC) (Han et al., 2017). Additionally, our previous study has demonstrated that miRNA plays a role in limiting the development of liver fibrosis by markedly blocking the activation and proliferation of hepatic stellate cells (HSCs), suggesting that miRNAs might be involved in the development and progression of several forms of cancers (Yang J. et al., 2017; Yang et al., 2019). Of note, lncRNAs, which are mainly transcribed by RNA polymerase II, are a new kind of ncRNA that are longer than 200 nucleotides (Ma et al., 2016). Owing to the lack of open reading frames, lncRNAs have extremely limited or no protein coding capacity (Ruan et al., 2016; Li J. et al., 2017). These new regulators were initially regarded as transcriptional noise with no specific biological functions (Kim and Sung, 2012). Recently, our laboratory found that epigenetic silencing of lncRNA ANRIL promoted the progression of liver fibrosis, thereby indicating that lncRNAs were associated with the progression of cancers (Yang et al., 2020). Interestingly, increasing evidence have shown that cellular events, including differentiation, proliferation, invasion, apoptosis, and migration, have all been associated with lncRNAs (Guttman et al., 2011). Additionally, there has been new evidence suggesting that lncRNAs may regulate a variety of biological and disease processes, from gene transcription and translation to post-translational modifications (Davalos and Esteller, 2019; Pang et al., 2019). More importantly, lncRNAs have been reported to be used as tumor suppressor genes or oncogenes, thus affecting the proliferation and metastasis of various types of tumors during tumorigenesis (Chen Q. N. et al., 2017; Lu et al., 2017). Subsequent studies have demonstrated that lncRNAs may serve as ceRNAs for miRNAs and in chromatin remodeling during the development of cancers (Huang et al., 2019; Wang C. J. et al., 2019). Figure 1 illustrates the functions of lncRNAs at the molecular level. Regarding certain cancer-associated human lncRNAs, it was demonstrated that linc-ROR was demonstrated to be predominantly upregulated in tumors (Peng et al., 2017a). The abnormal expression of linc-ROR in tumors has been suggested to be one of the main leading factors driving the development. The main ways to revert this effect would be to affect cell growth, migration, and invasion, thus leading to the inhibition of epithelial-mesenchymal transition (EMT), enhancement of the sensitivity to chemotherapy, etc. (Chen et al., 2016a; Zhao et al., 2017). For example, the expression level of linc-ROR in HCC tissues was inhibited compared with the adjacent tissues. At the same time, the downregulation of linc-ROR was linked to the aggressive process of the disease in patients with HCC. Furthermore, the ability of migration and invasion of HCC cells may be delayed by the low expression level of linc-ROR. In this review, we attempted to introduce the latest research on the biological effects, potential clinical applications, and molecular mechanisms of linc-ROR in human tumors and discuss its prognostic and therapeutic values.

## Overview of Linc-Ror

Among lncRNAs, linc-ROR is a novel and important carcinogenic 2.6 kb lncRNA located in chromosome 18, which was initially identified as a highly expressed transcript of pluripotent and embryonic stem cells (Chen et al., 2016b). Studies found that the octamer-binding transcription factor 4 (OCT4), SRY-box transcription factor 2 (SOX2), and Nanog homeobox (Nanog) key pluripotency factors could regulate linc-ROR (Wang et al., 2013). However, linc-ROR was also found to be expressed in several organs including lung, liver, breast, and colon. Since its discovery, research in this field has been extensively expanded during the past 6 years, revealing the important role of linc-ROR in tumorigenesis. Additionally, upregulation of linc-ROR has been suggested to mediate the re-expression of fetal and cardiomyocyte hypertrophy-related genes (Wang et al., 2013; Li et al., 2016). Many reports regarding linc-ROR and tumorigenesis in recent years, have revealed that the upregulation of linc-ROR is positively correlated with the clinicopathological characteristics and poor prognosis of tumors, including the stages of advanced tumor node metastasis (TNM), positive lymph node metastasis (LNM), and lower survival rate but higher recurrence rate.

**FIGURE 2:**
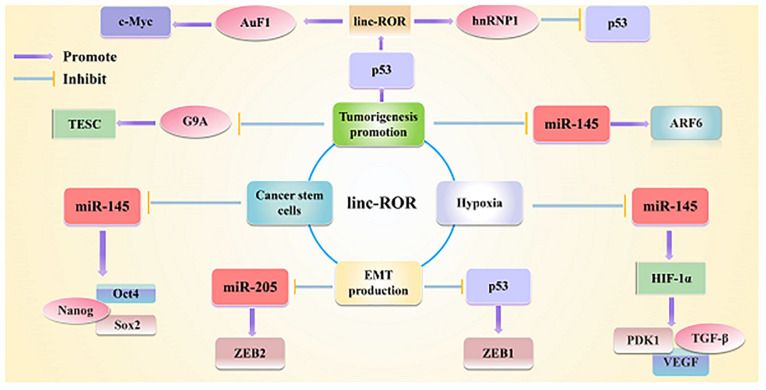
Linc-ROR is a typical lncRNA that plays important regulatory roles in interacting with miRNAs and maintaining stem cell pluripotency, as well as triggering the EMT as well. Linc-ROR is also involved in various key roles under various stresses and in epigenetic regulation.

Current evidence have strongly indicated that linc-ROR may exert an impact on a variety of cancers (Pan et al., 2016). Furthermore, both tumorigenesis and metastasis have been shown to be induced by linc-ROR *via* activation of the EMT in various cancers (Hou et al., 2014; Huang et al., 2016; Zhan et al., 2016). For example, linc-ROR was demonstrated to be upregulated, thereby promoting EMT in HCC (Li J. et al., 2017). Besides, it was also reported that self-renewal and differentiation of glioma stem cells was significantly affected by linc-ROR (Zhang et al., 2012; Feng et al., 2015). More importantly, the chemoresistance of pancreatic cancer (PC) and breast cancer (BC) (Li et al., 2016) as well as radio-resistance of colorectal cancer (CRC) cells were observed to be elevated by linc-ROR (Yang P. et al., 2017). Moreover, linc-ROR has also been shown to exert a significantly effect on the stem cell-like characteristics and tumorigenic potential of PC. Recently, it was also reported that linc-ROR could be used as a biomarker in the field of diagnosis and prognosis of BC and oral cancer (Arunkumar et al., 2017; Zhao et al., 2017). Notably, increasing studies showed that linc-ROR could be used as a ceRNA, thus exerting its impact in the post-transcriptional network of tumor pathogenesis. For example, in triple-negative BC, linc-ROR has been shown to serve as a ceRNA, therefore promoting the migration and invasion of BC cells (Signal et al., 2016). Overall, linc-ROR is a typical lncRNA that plays important regulatory roles in interaction with miRNAs and maintenance of stem cell pluripotency, triggering the EMT as well. Furthermore, linc-ROR has also been involved in various key roles under hypoxia and in the promotion of tumorigenesis (Figure 2). Therefore, linc-ROR may be considered as an oncogene affecting the progression of tumor and a promising predictor for the poor prognosis in patients with cancer. The transition of linc-ROR from basic research to clinical application requires further investigations as early as possible.

## Regulatory Role of Linc-Ror in Various Types of Cancer

**FIGURE 3:**
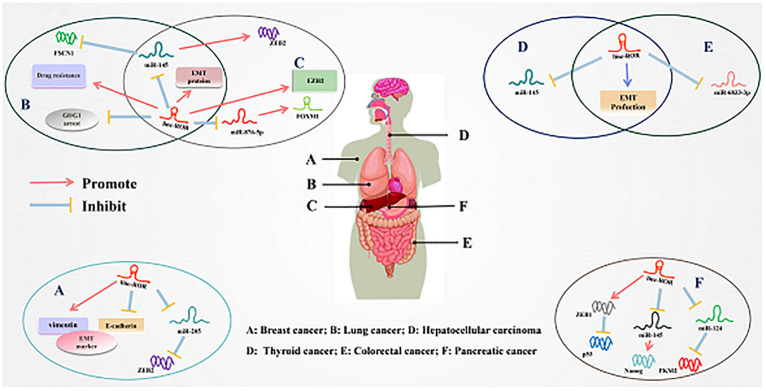
Underlying molecular mechanisms of linc-ROR in multiple cancers. **(A)** Linc-ROR binds to miR-205 to upregulate ZEB2, while it also regulated the expression of EMT markers. **(B)** Linc-ROR could interact with miR-145 to inhibit FSCN1 and upregulated EMT-associated proteins while it decreases G0/G1 arrest and facilitated drug resistance. **(C)** Linc-ROR facilitated EMT through upregulate EZHZ and regulated ZEB2 by competitively binding to miR-145, and ZEB2 overexpression leads to increased EMT. In addition, linc-ROR binds to miR-876-5p to upregulate FOXM1. **(D)** Linc-ROR downregulated through EMT production and repress the expression of miR-145. **(E)** Linc-ROR binds to miR-6833-3p, while also regulating the process of EMT. **(F)** Linc-ROR upregulated ZEB1 to attenuate the expression of p53, while also decreasing the expression of miR-145 to increase the level of Nanog and reduce that of miR-124 to suppress PKM2. Detailed mechanisms of linc-ROR in other cancers are provided in the review.

Increasing evidence has shown that the linc-ROR was abnormal expression in many cancers, and its dysregulation was associated with cellular functions (Fu et al., 2017; Spinelli et al., 2018). Additionally, studies found that the expression level of linc-ROR was substantially upregulated in samples of papillary thyroid carcinomas (PTCs) and PTCs cell lines as well as in metastatic PTCs samples and PTCs cell lines (Zhang et al., 2018). Simultaneously, cell migration and invasion could be regulated by linc-ROR *via* affecting EMT (Pastushenko and Blanpain, 2019). More importantly, studies demonstrated that linc-ROR was abnormally expressed in several cancers and led to elevated the invasion and metastasis of cancer cells to promoting the progression of tumors (Hashemian et al., 2019; Li et al., 2020b, c). This review summarizes the status of linc-ROR research in various human cancers and discusses its mechanism and clinical significance in the development and progression of tumor. The expression pattern, functional role, and regulatory mechanism of linc-ROR are summarized in Table 1 and depicted in Figure 3.

**TABLE 1:** Linc-ROR in human cancers.

**Cancer types**	**Expression**	**Role**	**Functional role**	**Clinical correlation**	**Regulatory molecules**	**References**
Breast cancer	Upregulated	Oncogenic	Proliferation, apoptosis, migration, invasion, EMT	TNM stage, LNM, ROC curve	linc-ROR/miR-205	Hou et al., 2018
Pancreatic cancer	Upregulated	Oncogenic	Growth, proliferation, migration, invasion, EMT	TNM stage, LNM, poor survival	linc-ROR/ZEB1/p53 linc-ROR/miR-145/Nanog linc-ROR/miR-124/PTBP1/PKM2	Gao et al., 2016; Li et al., 2016; Zhan et al., 2016
Hepatocellular carcinoma	Upregulated	Oncogenic	Poliferation, migration, invasion, EMT	TNM stage, LNM, DFS, OS	linc-ROR/EZH2/E-cadherin linc-ROR/miR-145/ZEB2, linc-ROR/miR-876-5p/FOXM1	Li C. et al., 2017; Chen et al., 2018; Zhi et al., 2019
Colorectal cancer	Upregulated	Oncogenic	Proliferation, apoptosis, migration, invasion, EMT, radiotherapy resistance.	TNM stage, LNM, DFS, OS	linc-ROR/miR-6833-3p	Yan and Sun, 2018; Li et al., 2020a
Lung cancer	Upregulated	Oncogenic	Apoptosis, cell cycle migration, invasion, EMT, drug resistance	TNM stage, LNM, DFS, OS	linc-ROR/miR-145/FSCN1	Pan et al., 2017
Thyroid cancer	Upregulated	Oncogenic	Proliferation, apoptosis, migration, invasion, cell cycle, EMT	TNM stage, LNM, poor survival	linc-ROR/miR-145	Zhang et al., 2018

### Breast Cancer

BC, which accounts for a quarter of all female cancer cases, is the most commonly diagnosed cancer and the leading cause of cancer-associated deaths among women worldwide (Li Z. et al., 2019). In 2018, it was estimated that there would be 2.1 million or so newly diagnosed cases of female BC (Bray et al., 2018; Hannafon et al., 2019). The main risk factors for BC, which is the difficult to change due to prolonged exposure to endogenous hormones, is difficult to control (Rudel et al., 2014; Bray et al., 2018). However, comprehensive treatment approaches have resulted in relatively good clinical outcomes for some patients with BC (Rudel et al., 2014; Goel et al., 2016; Kumler et al., 2016). Nevertheless, it has been reported that about one-third of the patients with BC have the potential for cell metastasis, chemotherapy resistance, and even recurrence (Goel et al., 2016; Kumler et al., 2016). Hence, there is an urgent need to develop new therapies targeting various molecular mechanisms of tumorigenesis for the treatment of BC.

Hou et al. investigated the expression level of linc-ROR in 94 patients (Hou et al., 2018). Their results revealed that the level of linc-ROR was increased in BC tissues. Moreover, the level of the expression of linc-ROR in the peripheral blood of the patients with BC was shown to be closely related to TNM phase and LNM. In addition, the wound-healing assay showed that overexpression of linc-ROR increased BC cells (MCF10A) mobility. Transwell assay revealed that linc-ROR overexpression remarkably increased the migration ability (Hou et al., 2018). More importantly, they found that ectopic expression of linc-ROR induced an EMT program in MCF10A cells. Fluorescence activated cell sorter analysis demonstrated that the subpopulation of the stem cell phenotype CD44^high^/CD24^low^ was elevated in MCF10A cells transfected with linc-ROR plasmid. Mechanistically, the results of bioinformatic analysis and RNA immunoprecipitation analysis from Hou et al. demonstrated that linc-ROR functions as a ceRNA to regulate miR-205 activity toward prevention of the degradation of transcripts of miR-205 target genes, such as ZEB1 and ZEB2, from degradation. Additionally, it was shown that the expression levels of miR-205 members were decreased upon linc-ROR overexpression in MCF10A cells (Hou et al., 2018). More importantly, Zhao et al. recently demonstrated that CRISPR/Cas9-generated BRCA1-knockdown adipose-derived stem cells stimulated a more aggressive behavior in BC cells than wild-type adipose-derived stem cells (Zhao et al., 2019). Therefore, we believe that CRISPR/Cas9 may be used to in the treatment of BC by inhibiting the expression of linc-ROR during the progression of BC. Conclusively, the linc-ROR/miRNAs axis has been reported to closely affect the occurrence and development of BC.

### Pancreatic Cancer

PC is the fourth most common cause of cancer-related mortality worldwide, leading to approximately 227,000 deaths annually (Siegel et al., 2016; Sabater et al., 2018). The 5−year relative survival of patients with PC remained at approximately 8% for 2005–2011 (Siegel et al., 2016). Hence, PC has been proposed to be one of the top two cancers in terms of fatalities in the next decade (Rahib et al., 2014). Surgical resection remains the exclusive potential curative treatment (Xiong et al., 2017). However, approximately half of the patients present with metastasis at the time of diagnosis, missing the opportunity for an effective treatment (Vincent et al., 2011; Xiong et al., 2017). A growing body of literature has demonstrated that both metastasis and limited effective biomarker for the diagnosis and treatment are the main obstacles for the efficient medical therapy of PC (Vincent et al., 2011; Boj et al., 2015; Basuroy et al., 2018). Thus, it is an absolute necessity to identify potential biomarkers and therapeutic targets in PC.

Zhan et al. (2016) have highlighted the oncogenic effects of linc-ROR in the initiation and progression of PC. Their study demonstrated that the level of linc-ROR was significantly elevated in PC tissues (Zhan et al., 2016). Moreover, the wound-healing assay and Boyden’s chamber assay results showed that linc-ROR silencing reduced the migratory capability and metastasis of PC cells (Zhan et al., 2016). Another study by Chen et al. showed that the proliferation rates of sh-ROR-cells in which the level of linc-ROR was suppressed were evidently lower than those of sh-NC-cells. This result was confirmed by colony formation assay, suggesting that linc-ROR accelerated the growth of PC cells (Chen et al., 2016a). Interestingly, silencing of linc-ROR was shown to result in increased levels of the epithelial markers E-cadherin and α-catenin, and decreased levels of mesenchymal markers N-cadherin and vimentin, indicating that linc-ROR plays an important role in the regulation of EMT in PC cells (Zhan et al., 2016; Chen et al., 2020). More importantly, microarray analysis identified ZEB1 as potential target gene of linc-ROR. Further, the expression of linc-ROR and ZEB1 were observed to be negatively correlated with that of p53, suggesting that linc-ROR might mediate migration, and metastasis in PC cells may partly *via* activation of ZEB1 through the inhibition of the expression of p53 (Zhan et al., 2016). Interestingly, the fluorescence *in situ* hybridization and luciferase reporter assay results showed that the expression of linc-ROR was demonstrated to be negatively correlated to that of miR-145. MiR-145 can induce posttranscriptional silencing of its targeted genes by binding to the mRNA 3’-UTR or linc-ROR specific sites, indicating that linc-ROR can act as a ceRNA to decrease miR-145 in PC cells, thereby activating expression of Nanog, thus leading to the proliferation of pancreatic cancer stem cells (PCSCs) (Gao et al., 2016). Additionally, Li et al. (2016) further proved that the impact of linc-ROR can be partly reversed by overexpression of miR-124. Consistently, linc-ROR was observed to exhibited a negative correlation with the expression of miR-124 (Li et al., 2016). Hence, a linc-ROR/miR-124/PTBP1/PKM2 axis was identified in PC, shedding new light on the lncRNA-based diagnosis and therapeutic approaches in PC (Li et al., 2016, 2020b). Notably, recent studies showed that PC cell-derived EVs could be used as effective carriers of paclitaxel to their parental cells, thereby bringing the drug into cells through an endocytic pathway and increasing its cytotoxicity (Saari et al., 2015). Additionally, it was demonstrated that vesicle-containing ncRNAs could serve as EV-associated PC detection markers (Worst et al., 2019). Thus, the presence of linc-RORs in EVs from patients with PC could serve as a potential diagnostic biomarker and a novel target for the therapy of patients with PC; this is worthy of further and wider research attention.

### Hepatocellular Carcinoma

As the sixth most international commonly occurring cancer in 2018, HCC has become the fourth cause of cancer-associated deaths worldwide. It has been estimated that 841,000 new cases and 782,000 deaths will occur each year (Bray et al., 2018). Briefly, HCC has been reported to account for 75–80% of all the liver cancer cases, half of which have been detected in China (Omata et al., 2017; Bray et al., 2018). As such, HCC poses a huge threat to the worldwide health, especially that of the Chinese people (Omata et al., 2017). About 70% of the patients is expected to recrudescent within 5 years after hepatectomy, and 30% of the patients will die from this tumor (Vigano et al., 2015). Therefore, on the basis of studying the pathogenesis of HCC, it is apparent to look for more effective molecular markers and therapeutic targets for the management of HCC.

Li C. et al. (2017) and Chen et al. (2018) reported that the expression level of linc-ROR was obviously elevated in 20 HCC tissues and four cell lines compared to the corresponding non-tumor tissues and normal liver cell lines, respectively, suggesting that linc-ROR might be critical regulator in the progression of HCC. Furthermore, biological function assay demonstrated that linc-ROR could play promoting role in regulating migration and invasion of HCC (Chen et al., 2018). Moreover, downregulation of linc-ROR could result in a significant increase in G1/G0 phase and an obvious decrease in S phase (Li C. et al., 2017). More importantly, silencing of linc-ROR could lead to the increased expression of E−cadherin and decreased expression level of N−cadherin in HCC cell lines. Li C. et al. (2017) further confirmed that linc-ROR could bind to the zeste homolog 2 (EZH2), thereby affecting the expression of E-cadherin, further indicating that linc-ROR could regulate the progression of EMT. Moreover, linc-ROR was further determined to be associated with DNA repair. Currently, mounting studies have identified reliable indicators of DNA damage, such as phosphorylated histone H2AX (γ-H2AX). Chen et al. (2018) uncover that overexpression of linc-ROR could obviously decrease the expression level of γ-H2AX, illuminating the suppressive effects of the overexpression of linc-ROR on DNA repair in HCC.

Further research demonstrated that linc-ROR could interact with miR−145 and dramatically downregulate the expression of miR−145 in HCC cells (Li C. et al., 2017). The above-mentioned results revealed that linc-ROR might play a promoting role in the proliferation, migration, invasion, and EMT of the HCC cell, which was contrary to the influence of miR−145 enrichment (Li C. et al., 2017). It suggested that overexpressed miR−145 could effectively reverse the promotion of HCC tumorigenesis induced by the overexpression of linc-ROR (Li C. et al., 2017). Li and his colleagues proposed a mechanistic model that linc-ROR promotes HCC tumorigenesis and autophagy partly through negatively regulating the expression of miR−145. Aside from that, it has been reported that miR-145 represses EMT, tumor migration, and invasion by directly targeting the 3’-UTRs of ZEB2 in the tumor. The decrease in miR−145 and increase in ZEB2 can obviously reversed the inhibition of cell migration and invasion mediated by the linc-ROR knockdown. Therefore, it was suggested that targeting the linc-ROR/miR−145/ZEB2 axis might represent a novel therapeutic application in HCC (Li C. et al., 2017). Similarly, Zhi et al. (2019) similarly showed that the migration and invasion of cells was reduced by the knockdown of linc-ROR. Moreover, they further confirmed that FOXM1-mediated activation of linc-ROR contributes to the poor sensitivity of HCC cells to sorafenib *via* partially regulating the miR-876-5p/FOXM1 axis, which forms a positive-feedback loop. Further evaluation of the regulatory mechanism involving this axis may provide new insights for exploring a potential therapeutic strategy for the management of HCC (Zhi et al., 2019). Consequently, these studies may offer new insights regarding the pathology of HCC and provide potential strategies for lncRNA-directed treatment. However, both the *in vivo* influence and other underlying mechanisms of linc-ROR still remain to be determined and clarified in the future research.

### Colorectal Cancer

There are approximately 1.3 million new CRC cases and 690,000 CRC-related deaths worldwide each year, thus making CRC the third most common cancer in the world (Torre et al., 2015). Although the treatment of CRC has significantly improved in recent decades, the prognosis remains unsatisfactory, especially in case of advanced tumors with distant metastases (Bogousslavsky, 1984; Torre et al., 2015). Current studies results showed that approximately 25% of cases with CRC have synchronous liver metastases during the time of diagnosis (Kawaguchi et al., 2019). These patients have inherently low survival rates of less than 10% within 5 years, with an even worse prognosis (Hu et al., 2019; Kawaguchi et al., 2019). Thus, there is an urgent need to better understand the progression of CRC and to identify novel and sensitive biomarkers for the diagnosis and treatment of patients with CRC.

Yang et al. detected the expression of linc-ROR in 30 CRC tissues compared to normal tissues by using qRT-PCR. They found that the expression of linc-ROR was remarkably increased in CRC tissues compared with normal tissues. Similarly, linc-ROR was shown to be overexpressed in five CRC cell lines (Yan and Sun, 2018; Li et al., 2020a). Then, they also performed a series of functional assays to clarify the biological effects of the aberrant expression of linc-ROR on proliferation, viability, apoptosis, migration, and invasion of CRC cells. Knockdown of linc-ROR was shown to effectively inhibit the proliferation of CRC cells, whereas its overexpression obviously increased the proliferative capacity of CRC cells. Accordingly, silencing of linc-ROR strongly inhibited the migratory and invasive abilities of CRC cells, compared with that in the control cells (Li et al., 2020a). In contrast, the migratory and invasive ability of cells was activated following the overexpression of linc-ROR. The MTS (3-(4,5-dimethylthiazol-2-yl)-5-(3-carboxymethoxyphenyl)-2-(4- sulfophenyl)-2H-tetrazolium) assay results showed that the overexpression of linc-ROR could enhance the viability of CRC cells. Furthermore, the flow cytometric analysis results revealed that the percentage of apoptotic cells in linc-ROR overexpression group was reduced by 9.74% ± 2.13%, indicating that the overexpression of linc-ROR could inhibit apoptosis in the CRC cell lines (Li et al., 2020a). More importantly, a recent study revealed the role of linc-ROR in the EMT. It was revealed that the upregulation of linc-ROR could increase the expression of N-cadherin and Vimentin as well as decrease the level of E-cadherin, leading to the promotion of the progression of EMT (Zhou et al., 2016; Yan and Sun, 2018). Meanwhile, the high expression of linc-ROR in CRC was also confirmed by Hu et al. (2019). Mechanistically, Li et al. (2020a) proved that that linc-ROR could bind to miR-6833-3p, which was determined to be significantly downregulated in CRC tissues. Additionally, a negative correlation was exhibited between the expression of linc-ROR and miR-6833-3p in BC tissues. Anti-AGO2 RNA immunoprecipitation assay further confirmed these results (Li et al., 2020a). Besides, rescue assays demonstrated that downregulation of miR-6833-3p could partly reversed the inhibition of tumorigenesis induced by linc-ROR knockdown in BC cells. Li and his colleagues uncovered that linc-ROR exerted its oncogenic role through negatively regulating the expression of miR-6833-3p during the progression of CRC, which might give new insights into molecular diagnosis and treatment (Li et al., 2020a). In addition, Li et al. (2020a) further uncovered that linc-ROR could mediate the expression level of SMC by sponging miR-6833-3p in CRC cells, thus promoting CRC progression. As for the effects of linc-ROR on radiotherapy resistance. Yan and Sun (2018) showed that overexpression of linc-ROR increased the ability of CRC cells for radiotherapy resistance. Collectively, these findings indicated that linc-ROR might be engaged in the metastatic process of CRC cells and could promote the development of CRC through a variety of molecular mechanisms.

### Lung Cancer

Lung cancer is the leading cause of cancer-related deaths worldwide (Bray et al., 2018). Non-small cell lung cancer (NSCLC) accounts for about 85% of the lung cancer types, including squamous cell carcinoma, large cell lung cancer, and lung adenocarcinoma (Herbertz et al., 2015; Bray et al., 2018; Brainard and Farver, 2019). Although there are various approaches for its diagnosis and treatments, the 5-year overall survival (OS) rate for patients with advanced lung cancer is less than 15% (Zhou et al., 2011). Therefore, in order to carry out an early diagnosis and treatment of lung cancer, the search for valuable and efficient tumor markers is very urgent.

In recent years, linc-ROR has appeared as an important regulator of lung cancer. Research from Qu et al. (2017) demonstrated that the expression of linc-ROR was increased in 299 NSCLC tissues compared to that in the normal tissues. The overexpression of linc-ROR was shown to be closely related to the poor prognosis of LNM, histological grade, and stage of TNM (Pan et al., 2017; Qu et al., 2017). Another study by Pan et al. (2017) demonstrated that the decreased expression of linc-ROR could obviously impair the proliferative capacity of lung adenocarcinoma (LAD) cells, cause a G0/G1 phase arrest, and increase the ratio of apoptotic LAD cells. Moreover, downregulation of linc-ROR substantially inhibited the invasive and metastatic ability of LAD cells. Meanwhile, forced expression of linc-ROR was observed to reduce the expression of E-cadherin and β-catenin, which are the characteristic biomarkers of epithelial cells, whereas it increased the expression of N-cadherin and Vimentin, thus displaying a mesenchymal phenotype. Conversely, downregulation of linc-ROR was demonstrated to result in increased the expression of epithelial markers and decreased the expression of mesenchymal markers. This result uncovered the fact that the pro-metastatic effects of linc-ROR were induced by the regulation of the expression of a number of genes involved in cell metastasis and EMT progress. Meanwhile, overexpression of linc-ROR was shown to enhance the resistance of LAD to docetaxel (DTX) (Pan et al., 2017), suggesting that linc-ROR-induced resistance of LAD cells to DTX and EMT through regulation the expression of miR-145, which was predicted to interact with linc-ROR. More importantly, further research confirmed that miR-145 could bind to linc-ROR, and its downregulation could partly inhibit the resistance of LAD cells to DTX and EMT. Aside from that, Pan and his colleagues discovered that a decrease in the expression of miR-145 and increase in the expression FSCN1 could obviously reverse the inhibition of cell proliferation, chemoresistance, and EMT mediated by linc-ROR knockdown. They identified that dysregulation of the linc-ROR/miR-145/FSCN1 axis was associated with the therapeutic resistance and EMT transition in LAD cells, thereby providing potential therapeutic strategies for managing drug resistance in patients with LAD (Pan et al., 2017). Taken together, these studies collectively suggested that linc-ROR could activate the malignant phenotype of NSCLC cells with the guidance of a mechanism involving miRNAs. Hence, more efforts should be made to elucidate other regulatory mechanisms and the clinical significance of linc-ROR in lung cancer.

### Thyroid Cancer (TC)

TC continues to be the most common endocrine malignant tumor and has emerged as a major health issue (Ito et al., 2013). It is estimated that more than 60,000 people in the United States present with TC every year (Ito et al., 2013; Vigneri et al., 2015). Additionally, TC is the sixth most common malignancy among Chinese women, with an incidence rate of about 6.6 per a population of 100,000 (Chen et al., 2015). The major subtypes of TC include PTC, follicular thyroid cancer (FTC), poorly differentiated thyroid cancer (PDTC), and anaplastic thyroid cancer (ATC) originating from follicular cell-derived thyroid cells. PTC accounts for more than 85% of all the TC cases, and approximately 10–15% of the patients with PTC have been reported to exhibit relapse and metastasis after therapy, leading to a poor outcome (Fagin and Wells, 2016). On the other hand, ATC is the most aggressive and fatal subtype, with a total survival of merely 3–5 months after initial diagnosis (Fagin and Wells, 2016). The studies of molecular mechanism correlated with the development and progression of TC may considerably facilitate the understanding of the pathogenesis of TC (Wang et al., 2016, 2017; Murugan et al., 2018). Thus, it is importantly to find potential biomarkers and therapeutic targets involved in TC tumorigenesis.

Zhang et al. (2018) examined the expression levels of linc-ROR in TC cells. Accordingly, their results from *in situ* hybridization showed that the levels of linc-ROR was increased in TC tissues and TC cell lines. Then, the expression of linc-ROR was further validated by using qRT-PCR analysis in TC tissue samples and cell lines, as they found it to be higher compared to that in normal tissue samples and normal thyroid cell. Hence, linc-ROR was observed to be evidently upregulated during the progression of TC (Zhang et al., 2018). Then, a series of functional assays were performed to clarify the biological effects of linc-ROR on proliferation and invasion of TC cells. They showed that that decreased the expression of linc-ROR could suppress the proliferative and invasion capacity of TC cells, as well as the elevated apoptotic rate in TC cells (Zhang et al., 2018). Interestingly, this led to the reversed progress of EMT, which was presented as a reduction in the levels of E-cadherin and enrichment in those of N-cadherin, highlighting the involvement of linc-ROR in the regulation of EMT (Zhang et al., 2018). Specifically, Zhang et al. (2018) proved that linc-ROR could act as a molecular sponge to modulate miR-145, which was determined to be significantly downregulated in TC tissues, indicating that linc-ROR had a negative regulatory role on miR-145. In summary, Zhang et al. discovered that linc-ROR could act as an oncogene and suggested its utilization as a prognostic indicator of patients with TC. However, a larger cohort of tumor samples and deeper mechanistic research are urgently needed.

## Potential Clinical Application of Linc-Ror in Human Cancers

The detection of cancer is hard in early stages and thus losing the best chance for curative surgery results in the poor survival rates (Siegel et al., 2020). Specific cancer biomarkers that monitor the molecular differences associated with cancer, which may be used for early diagnosis, are essential and may help select the best treatment options and gain valuable treatment time for patients with cancer. In clinical settings, prognostic markers can be used to predict the clinical outcome of untreated patients with cancer. On the other hand, current studies have further indicated that the progression of cancer or differentiation of cancer subtypes can be predicted by the expression profiles of lncRNAs. However, because of the uncertain molecular function of lncRNAs, it is difficult to accurately predict the progress of tumors. The ideal and convenient biomarkers should possess several typical and important characteristics. Interestingly, studies have shown that linc-ROR is not only closely associated with multiple biological functions of cancer cells but may also be an ideal and convenient biomarker (Pan et al., 2016). For example, the expression of linc-ROR has been reported to be increased in HCC tissues with LNM or vascular infiltration compared with normal tissues. In addition, the advanced stage of TNM was associated with the overexpression of linc-ROR. Most importantly, the expression of linc-ROR was shown to be elevated in patients with HCC with recurrence compared with those without recurrence (Li C. et al., 2017). Furthermore, results obtained from the Kaplan-Meier analysis showed that patients with HCC with high expression of linc-ROR had worse prognosis compared to those with low linc-ROR expression, along with a shorter disease-free survival (DFS) and OS (Li C. et al., 2017).

Zhao et al. (2017) found that the expression level of linc-ROR was increased in BC tissues, BC cell lines, and BC plasma samples. Concomitantly, they demonstrated that the level of linc-ROR in patients with LNM was elevated compared to that in patients without LNM. Besides, the expression of linc-ROR in ER-positive or PR-positive BC plasma also demonstrated to be significantly increased (Zhao et al., 2017). Moreover, the study showed that the area under the Receiver operating characteristic (ROC) curve (AUC) of linc-ROR was 0.758 (sensitivity 80.0%; specificity 73.3%), suggesting that the diagnostic ability of linc-ROR was elevated relative to that of Carcinoembryonic antigen (CEA) (AUC = 0.516; sensitivity 66.7%; specificity 50.0%) and CA153 (AUC = 0.663; sensitivity 73.3%; specificity 60.0%). More importantly, they found that linc-ROR had the strongest ability among the three indices in distinguishing healthy from BC-affected individuals in an evaluation of 45 early stage patients from the 96 patients with BC and 45 age and sex-matched healthy controls from the 90 healthy volunteers. Moreover, the combined detection of the three plasma indexes might enhance the overall diagnostic power (AUC = 0.846; sensitivity 83.3%; specificity 70.0%) (Zhao et al., 2017). In addition, Hou et al. (2018) examined the level of linc-ROR in the clinical prognosis of patients with BC. The group found that there was a close relationship between LNM and the expression of linc-ROR. More importantly, overexpression of linc-ROR was observed to exhibit a faster decline with increased survival (Chen et al., 2016a; Hou et al., 2018). Another study also revealed that the regulatory polymorphisms in linc-ROR had an impact on the risk for BC (Luo et al., 2018). Conclusively, all the above-mentioned results suggested that linc-ROR might be used as a diagnostic and prognostic biomarker for BC.

Qu et al. (2017) identified that the level of linc-ROR was significantly elevated in NSCLC tissues compared to that in the adjacent tissues. Meanwhile, upregulation of linc-ROR was shown to be significantly linked to advanced TNM stage, LNM, and positive distant metastasis. The Kaplan-Meier curve analysis showed that patients with NSCLC with high expression of linc-ROR have shorter OS and DFS times compared to patients with low expression of linc-ROR (Duan et al., 2016; Jiang et al., 2016; Qu et al., 2017). Subsequently, the results of Cox proportional hazards regression analyses showed that the expression of linc-ROR was an independent prognostic indicator for OS (HR (heart rate) = 2.983) and DFS (HR (heart rate) = 3.421) in patients with NSCLC (Qu et al., 2017). In addition, Fu et al. (2017) also demonstrated that the expression level of linc-ROR was increased in PC tissues compared to that in adjacent tissues. The expression level of linc-ROR was further demonstrated to be closely linked to tumor size and the clinical stage of PC. Meanwhile, log-rank analysis indicated that the OS was significantly decreased in patients with higher expression of linc-ROR (Fu et al., 2017). Glioblastoma (GB) has been considered to be the most aggressive subtype of glioma and the most common adult malignant brain tumor in the world (Stupp et al., 2009; Linz, 2010; Davis, 2016). Toraih et al. (2019) reported that the level of linc-ROR was increased in 89.5% of the patients (Han et al., 2012; Feng et al., 2015). The overexpression of linc-ROR was observed to be closely linked to poor disease progression-free and OS in younger age of patients. In addition, Kaplan–Meier survival plot illustrated that patients with GB with high linc-ROR expression had low survival rates. Multivariate analysis demonstrated that patients with GB were divided into two different groups according to the expression profile of linc-ROR and OS of patients (Toraih et al., 2019). Thus, these results suggested that the expression of linc-ROR was complementary in playing a crucial role in the progress of cancers and thus could serve as a new potential biomarker for the evaluation of clinical prognosis.

Currently, the biggest obstacles to cancer treatment are delayed diagnosis, recurrence, and metastasis. Therefore, the search for the ideal cancer biomarkers is crucial for the improvement of the early diagnosis rate. The above-mentioned findings suggested that linc-ROR might be used as a potential marker for the diagnosis of several types of cancer. However, the exact molecular mechanism by which linc-ROR plays a role in various cancers remains unclear. As such, the functional role of linc-ROR in cancer needs to be further explored and verified, especially in clinical applications.

## Regulatory Mechanism of Linc-Ror in Various Types of Cancer

It is well understood that improvement of the survival of patients requires a deeper understanding, as well as effective biomarkers for determination of cancer occurrence and metastasis. Additionally, detection of these invasive tumors in the early stages of disease development and development of efficient treatments are more effective ways to reduce cancer mortality. A large number of researches have shown that many signaling pathways were significantly associated with the occurrence and development of tumors (Chen et al., 2016a; Sahebi et al., 2016; Hou et al., 2018). Hence, we summarize that the related some regulatory mechanisms are associated with the tumor development and progression.

**FIGURE 4:**
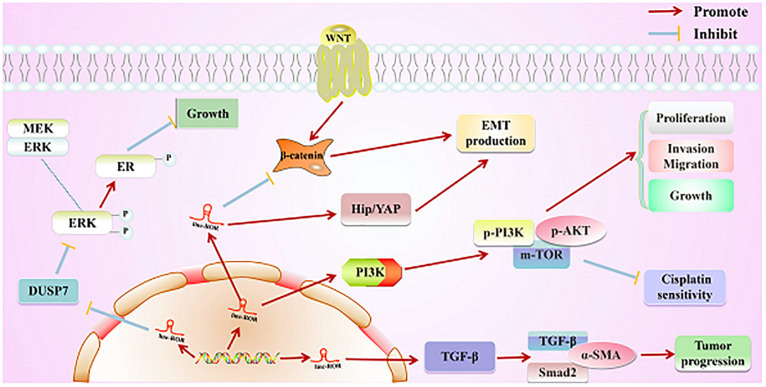
Direct regulatory function of linc-ROR in various cancers. First, linc-ROR promotes the estrogen-independent growth and activation of the MAPK/ERK signaling pathway of tumor cells by regulating the DUSP7 ERK-specific phosphatase. Second, linc-ROR promotes the process of EMT through the Wnt/β-catenin and Hip/YAP signaling pathways. Additionally, linc-ROR strongly represses the sensitivity to cisplatin and significantly increases the proliferation, invasion, migration, and growth of tumor cells *via* the PI3K/AKT/m-TOR signaling pathway (associated proteins: p-PI3K, p-AKT, and p-mTOR). Simultaneously, linc-ROR significantly facilitates the progression of tumor through the TGF-β signaling pathway (associated proteins: p-PI3K, p-AKT, and p-mTOR).

First, linc-ROR has been reported to directly affect the progression of various cancers through several signaling pathways (Figure 4 and Table 2). Of note, a number of studies have hinted that the aberrant activated MAPK/ERK cascade plays a critical role in many aspects of tumorigenesis. Nearly 50% of human malignancies are known to exhibit unregulated ERK signaling (Herrero et al., 2015). In BC, the upregulated MAPK/ERK (mitogen-activated protein kinase/extracellular regulated protein kinases) signaling has been correlated with poor survival in patients with triple-negative BC (Bartholomeusz et al., 2012). In addition, the MAPK/ERK pathway has also been shown to influence the chemotherapeutic drug resistance to doxorubicin and paclitaxel in BC cells (McCubrey et al., 2006). Moreover, It is well known that the MAPK/ERK signaling is negatively regulated by mitogen-activated protein kinase phosphatases, which form six distinct groups based on their physiological functions; among them, groups 1–4 are known to be involved in dephosphorylation of ERK, including DUSP7 (Owens and Keyse, 2007). Interestingly, Peng and his colleagues demonstrated that DUSP7 is downregulated in response to estrogen deprivation and overexpression of linc-ROR significantly decreased the half-life of DUSP7, possibly inhibiting the activation of ERK, resulting in estrogen-independent growth of BC cells (Peng et al., 2017a). The Wnt signaling cascade was highly conserved among species and controls a multitude of biological processes during animal development and life cycles. Because of its central role in the maintenance of tissue homeostasis, the Wnt pathway was tightly regulated at multiple levels, from the ligand-receptor interaction down to transcriptional and post-transcriptional levels; its aberrant activity has been implicated in a number of developmental disorders and diseases and, most prominently, in cancer (Nusse and Clevers, 2017; Nguyen et al., 2019; Zhang et al., 2019; Liu et al., 2020). The recent discovery of lncRNAs that are regulated by Wnt and/or participate in Wnt pathway modulation and outcome is particularly intriguing and has highlighted some of these gaps in our knowledge (Zarkou et al., 2018). For example, Lou et al. (2017) have discovered that the Wnt/β-catenin signaling pathway led to the elevated proliferation of ovarian cancer (OC) cells. Whereas, E-cadherin was shown to be decreased, vimentin, β-catenin, and c-myc were all increased in OC cells treated with LiC1 (a Wnt/β-catenin pathway activator) compared with untreated controls (Lou et al., 2017). More importantly, the study further revealed that the activation of Wnt/β-catenin signaling pathway and the progression of EMT, along with the abnormal expression of EMT markers and Wnt/β-catenin signaling pathway-related proteins (c-Myc, cyclin-D1, and β-catenin), was inhibited by knockdown of linc-ROR during the development of OC, indicating that linc-ROR promotes OC EMT at least in part by activating the Wnt/β-catenin pathway (Lou et al., 2017). However, the relationship between linc-ROR and the Wnt/β-catenin signaling pathway required deeper study in tumor initiation and progression. The PI3K/Akt/mTOR pathway has various cell functions, including cellular proliferation, survival, differentiation, and intrusion, and previous literature has proven that lncRNA silencing could reduce the phosphorylation of ERK, Akt, and mTOR (Fumarola et al., 2014). More importantly, aberrant activation of the PI3K/Akt/mTOR pathway was shown to be one of the mechanisms of targeted therapeutic resistance in patients with NSCLC (Fumarola et al., 2014). Xu et al. (2018) found that silencing of linc-ROR significantly inhibited the expression of p-PI3K, p-AKT3, and m-TOR. In contrast, the overexpression of linc-ROR was shown to increase the expression of p-PI3K, p-AKT3, and m-TOR, suggesting that the activation of PI3K/Akt/mTOR signaling pathway was mediated by linc-ROR in NSCLC (Xu et al., 2018). Similarly, Shi et al. (2017) further discovered that linc-ROR could inhibit the PI3K/Akt/mTOR signaling pathway, so that inactivation of linc-ROR or inhibition of PI3K/Akt/mTOR signaling pathway could increase the sensitivity of NSCLC to cisplatin (DDP), thereby promoting cell apoptosis, and inhibiting cell proliferation, migration, invasion, and tumor growth. Additionally, the Hippo/YAP pathway (also known as the Salvador–Warts–Hippo pathway) is an evolutionarily conserved regulator of tissue growth and cell fate (Harvey et al., 2013). The Hippo/YAP pathway was first postulated to be important for human cancer on the basis of the egregious overgrowth of *Drosophila melanogaster* tissues that harbor mutations in different Hippo/YAP pathway genes. In recent years, increasing numbers of mammalian studies have validated this hypothesis: Hippo/YAP pathway perturbation can trigger tumorigenesis in mice, and mutation and altered expression of a subset of Hippo pathway genes have been observed in human cancers (Harvey et al., 2013). Interestingly, the Hippo/YAP signaling pathway has also been reported to activated by linc-ROR (Mo et al., 2014). Inactivation of the Hippo signaling pathway could lead to the downregulation of MST1/LATS1 (the core factors of Hippo pathway) and upregulation of YAP1 (He et al., 2015). Furthermore, dysregulation of the Hippo signaling has been confirmed in many types of tumors and closely linked to the acquisition of malignant features. A recent study provided by Chen et al. demonstrated that the regulatory axis of linc-ROR/EMT/YAP1 regulatory axis exerted oncogenic effects in the progression of PC (Chen et al., 2020). They found that a knockdown of linc-ROR resulted in the downregulation of YAP, and MOB kinase activator 1 (MOB1), whereas there was upregulation of MST1, MST2, p-MOB1, p-LATS1, LATS1, and p-YAP in PC cells. In contrast, overexpression of linc-ROR led to the exact reverse condition (Chen et al., 2020). The current evidence for the existence of the linc-ROR/EMT/Hippo/YAP axis has indicated that combined targeting of the YAP1/Hippo signaling pathway may provide a potential direction for the treatment of PC. Many studies have recognized that the function of TGFβ in the progression of BC can be different depending on the stage of cancer (Bachman and Park, 2005; Moses and Barcellos-Hoff, 2011; Massague, 2012). Under normal conditions, TGF-β has been shown to inhibit cell cycle and promote apoptosis, which together significantly contribute to its suppressive role in the initiation and progression of tumorigenesis (Heldin et al., 2009). Hou et al. (2018) reported that knockdown of linc-ROR in BC cells led to diminished expression levels of TGF-β. As a result, the downstream factors, such as Smad2 and α-SMA, were also downregulated. This finding suggested that overexpression of linc-ROR might be required to constitutively upregulate critical factors in the TGF-β signaling pathway (Hou et al., 2018). Overall, these findings revealed that linc-ROR might provide a novel therapeutic target and biomarker for cancers in the future.

**TABLE 2:** The involvement of linc-ROR in multiple signaling pathways.

**Cancer types**	**Role**	**Expression**	**Related gene**	**Signaling pathway**	**References**
Breast cancer	Oncogenic	Upregulated	ERK1, ERK2 TGF-β, Smad2, and α-SMA	MAPK/ERK signaling pathway TGF-β signaling pathway	Peng et al., 2017a; Hou et al., 2018
Pancreatic cancer	Oncogenic	Upregulated	MST1, MST2, p−MOB1, p−Lats1, Lats1, p−YAP	Hippo/YAP signaling pathway	Chen et al., 2020
Lung cancer	Oncogenic	Upregulated	p-PI3K, p-AKT3, m-TOR	PI3K/Akt/mTOR signaling pathway	Shi et al., 2017; Xu et al., 2018
Ovarian cancer	Oncogenic	Upregulated	c-Myc, cyclin-D1, and β-catenin	Wnt/β-catenin signaling pathway	Lou et al., 2017

## Future Prospects

A growing understanding of the role of linc-ROR in all types of cancers may opened up the possibility for many new treatment strategies. Therefore, increasingly, cancer researchers will focus on this lncRNA. Recent studies have displayed that the level of linc-ROR was increased in lung cancer, bladder cancer, and CRC cell lines. Moreover, both cell proliferation and migration have been reported to be promoted by linc-ROR in several types of cancers. In addition, increasing evidence have shown that the abnormal expression of linc-ROR in various cancers was closely linked to tumorigenesis, diagnosis, metastasis, and prognosis through some signaling pathways. Of note, a number of studies have confirmed that linc-ROR can be considered as a possible new target for cancer therapy and a biomarker for cancer diagnosis. However, the current knowledge on the biological functions of linc-ROR remains insufficient, and its efficacy is also not evident. Thus, additional studies on lncRNAs are required.

The abbreviation “EV” is actually a collective term that refers to a series of lipid bilayer membrane-bound organelles that are released by cells into their environment. Briefly, EVs are heterogeneous in size and released from nearly all cells under appropriate physiological and pathological conditions (Lee et al., 2011). Of note, a variety of cargos have been transferred from a cell to another cell *via* EVs (Ekstrom et al., 2012; Yang J. et al., 2018). More importantly, the EV cargo can reflect the cells of origin. A number of studies have demonstrated that exosomes carry different types of RNA compared to their parental cells (Skog et al., 2008; Ekstrom et al., 2012; Nolte-’t Hoen et al., 2012). It is well-known that unprotected ncRNAs are easily degraded by the RNases in the blood. However, it has been demonstrated that under the protection of EVs, ncRNAs avoid degradation, and thus their integrity and activity are maintained in the circulation. Recently, studies suggested that the expression of lncRNAs in the plasma of patients with HCC was considerably increased compared to that in healthy people. More importantly, Takahashi et al. assessed the role of EV signaling in tumor cell responses to TGF-β and also recognized specific EV-lncRNA mediators, such as linc-ROR, involved in the regulation of the chemotherapy response to chemotherapy (Takahashi et al., 2014; Spinelli et al., 2018). Targeting these intercellular signaling mechanisms and mediators may be useful in enhancing sensitivity and improving responses to conventional therapeutic agents that are used for the treatment of HCC. As such, the expression of linc-ROR, which plays an important role in cancer prevention, diagnosis, and treatment may be increased through the administration of exosomal linc-ROR.

On the other hand, CRISPR/Cas9, as a very powerful gene editing tool, has been successfully applied to the interruption of the protein-coding 24 sequence in various organisms (Li M. et al., 2019). Previous researches have demonstrated that CRISPR/Cas9 could regulate the progression and development of cancers. For example, CRISPR/Cas9-mediated synthesis and gate genetic circuits were shown to be efficiently employed in the identification of bladder cancer cells (Liu et al., 2014). Of note, CRISPR/Cas9 could successfully target lncRNAs and replace the overexpression of the sponge bait, thus negating the requirement for the introduction of transgenes (Kellner et al., 2018). In addition, CRISPR/Cas9 has achieved many successes as a powerful genome engineering tool for the treatment of many diseases due to its specificity, efficiency, simplicity, and versatility (Zhen et al., 2017). For example, both the transcription and infection by HIV-1 were shown to be increased by lnc-MALAT1. However, long terminal repeat-driven gene transcription of HIV-1 and viral replication were reported to be inhibited by CRISPR/Cas9-mediated lnc-MALAT1 knockdown. Similarly, knockdown of lnc-HTOR or IGF2BP1 by CRISPR/Cas9 gene-editing methods mimicked the effects and abolished the requirement for Triptonide in nasopharyngeal carcinoma cells (Liu et al., 2016). Therefore, there’s a good chance that the technology of CRISPR/Cas9 could be used to treat cancers by editing the expression level of linc-ROR. Thus, CRISPR/Cas9 might be used to treat cancers by regulating the expression of linc-ROR *via* the relevant molecular mechanism.

## Conclusion

Increasing studies have demonstrated that lncRNAs could regulate the expression of genes and be involved in the tumorigenesis and development of tumors through complex tumor networks. The linc-ROR was reported to be abnormally expression in a variety of malignant tumors, such as BC, PC, HCC, CRC, NSCLC, and TC. The expression trend of this lncRNA was demonstrated to be almost similar in most of these cancers. In addition, studies confirmed that the linc-ROR served as an oncogene *in vivo* and *in vitro* and was closely associated with the enlarged tumor volume, advanced TNM stage, shortened OS, and metastasis. Meanwhile, cell proliferation, invasion, migration, and anti-apoptosis were observed to be regulated by linc-ROR in all the above-mentioned cancers. Retrospective studies of linc-ROR in human cancers may provide new targets for the diagnosis and treatment of different types of cancer. Furthermore, increasing studies have shown that the molecular mechanism, such as Wnt/β-catenin signaling pathway involved in the progress of cancer, was regulated by linc-ROR. There seems to be some competitions between linc-ROR and miRNAs, resulting in the inhibition of downstream target genes of these miRNAs. However, the exact mechanism of action of linc-ROR remains to be explored. Further experiments are needed to delineate the role of linc-ROR and its molecular mechanisms in other human malignancies.

## Author Contributions

WC and JY drafted the manuscript. JS revised the manuscript. LL and HF reviewed and modified the manuscript. All authors agreed on the final version.

## Conflict of Interest

The authors declare that the research was conducted in the absence of any commercial or financial relationships that could be construed as a potential conflict of interest.

## Abbreviations

BC: breast cancer
ceNAs: competing endogenous RNA
EMT: epithelial–mesenchymal transition
HCC: hepatocellular cancer
iPSCs: induced pluripotent stem cells
linc-ROR: Long intergenic non-protein coding RNA, regulator of reprogramming
lncRNAs: Long non-coding RNAs
PC: pancreatic cancer.
